# Pyrogallol Structure in Polyphenols is Involved in Apoptosis-induction on HEK293T and K562 Cells 

**DOI:** 10.3390/molecules13122998

**Published:** 2008-12-04

**Authors:** Shinya Mitsuhashi, Akiko Saito, Noriyuki Nakajima, Hiroshi Shima, Makoto Ubukata

**Affiliations:** 1 Division of Applied Bioscience, Research Faculty of Agriculture, Hokkaido University, Kita-9, Nishi-9, Kita-ku, Sapporo 060-8589, Japan; E-mail: shinya@igm.hokudai.ac.jp (S. M.); 2 Biotechnology Center, Toyama Prefecture, Kosugi, Toyama 939-0398, Japan; 3 Present address: Antibiotics Laboratory, Discovery Research Institute, RIKEN 2-1 Hirosawa, Wako-shi, Saitama 351-0198, Japan; E-mail: saiaki@riken.jp (A. S.); 4 Biotechnology Research Center, Toyama Prefectural University, Kosugi, Toyama 939-0398, Japan; E-mail: nori@pu-toyama.ac.jp (N. N.); 5 Division of Cancer Chemotherapy, Research Institute, Miyagi Cancer Center, Natori, Japan; E-mail: shima-hi632@pref.miyagi.jp (H. S.)

**Keywords:** Cytotoxicity, Apoptosis, Pyrogallol, polyphenol, Structure-activity relationship.

## Abstract

As multiple mechanisms account for polyphenol-induced cytotoxicity, the development of structure-activity relationships (SARs) may facilitate research on cancer therapy. We studied SARs of representatives of 10 polyphenol structural types: (+)-catechin (**1**), (-)-epicatechin (**2**), (-)-epigallocatechin (**3**), (-)-epigallocatechin gallate (**4**), gallic acid (**5**), procyanidin B2 (**6**), procyanidin B3 (**7**), procyanidin B4 (**8**), procyanidin C1 (**9**), and procyanidin C2 (**10**). Amongst them, the polyphenols containing a pyrogallol moiety (**3**-**5**) showed the most potent cytotoxicic activity. These compounds evoked a typical DNA-laddering phenomenon in HEK293T, which indicated that the induction of apoptosis at least partly mediates their cytotoxic activity. Anti-oxidative capacity of compounds **3**-**5** were comparable to those of the trimers **9** and **10**, which were not cytotoxic. Therefore, we suggest that pyrogallol moiety is important for fitting of polyphenols to their putative target molecule(s) in non-oxidative mechanism.

## Introduction

Polyphenols such as the proanthocyanidins and catechins, are naturally occurring plant metabolites widely available in fruits, vegetables, nuts, seeds, flowers, and bark [1,2]. They can easily react with reactive oxygen species (ROS), resulting in powerful antioxidant activity. This property is partly responsible for the correlation between increased polyphenol intake and a reduced risk for cancer [3], stroke [4] and coronary heart disease [5]. Several polyphenols have been reported as apoptosis-inducers and inhibitors of cell proliferation in human tumour cells [6]. The relevant mechanisms of action are complex and may involve interaction with one or more cell components such as mitogen-activated protein kinases, DNA polymerase, cyclin-dependent kinases, activation of activator protein 1 (AP-1), nuclear factor kappa B (NF-κB) and growth factor signalling [7,8]. The impact of polyphenol on the ROS level is also of importance.

**Figure 1:**
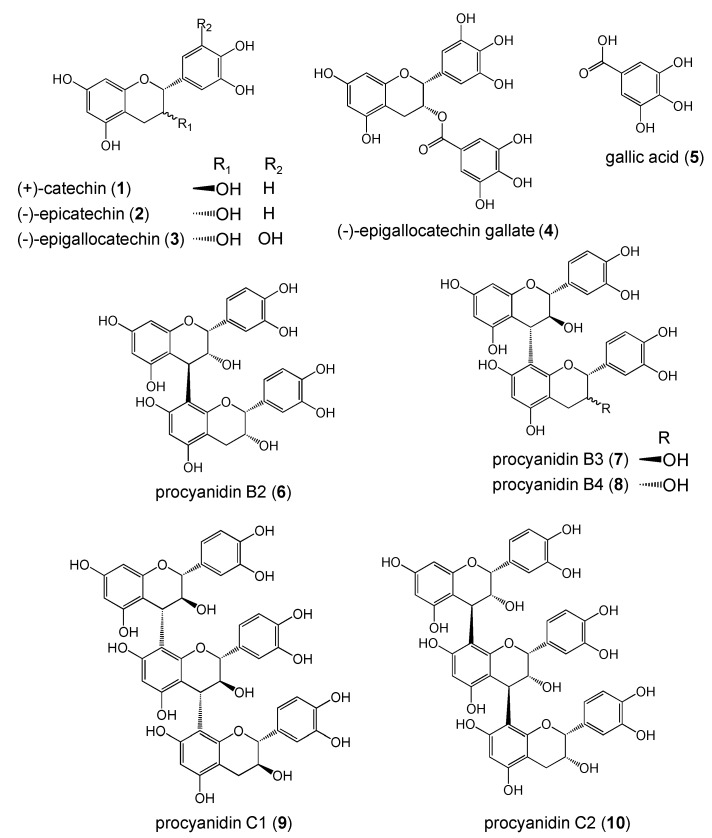
Structures of polyphenols.

Although polyphenols have beneficial effects on human health, SARs between polyphenols and cytotoxicity are not well understood. As multiple mechanisms account for polyphenol-induced cytotoxicity, the development of SARs to predict the cytotoxic potential of a given compound may facilitate the search for effective candidates for cancer therapy. Thus, to explore important structure for induction of cell death, we studied cytotoxicity and radical scavenging activity of 10 polyphenol structural types. 

## Results and Discussion

In this study, we used different polyphenols including (+)-catechin (**1**), (-)-epicatechin (**2**), (-)-epigallocatechin (**3**), (-)-epigallocatechin gallate (**4**), gallic acid (**5**), procyanidin B2 (**6**), procyanidin B3 (**7**), procyanidin B4 (**8**), procyanidin C1 (**9**), and procyanidin C2 (**10**) (Figure 1). These compounds have flavonoid structure except for **5**, and are classified as monomer (**1**-**4**), dimer (**6**-**8**), and trimer (**9**, **10**) types. 

**Figure 2:**
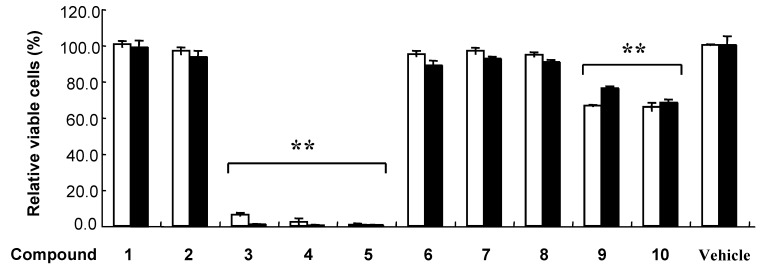
Effects of polyphenols on cultured cells.

K562 (open bar) and HEK293T (closed bar) were incubated with 100 μM polyphenol (**1**-**10**) or 0.5 % DMSO (Vehicle) for 48 h. Error bar indicates standard deviation. The double asterisk (**) indicate significant difference (*P* < 0.01) by the Scheffe’s multiple comparison test to the control cells treated with DMSO.

**Table 1:** Cytotoxic activities of compound **3**-**5** on cultured cells.

	K562			HEK293T		
	LC_50_ (μM)			LC_50_ (μM)		
Compound	24 h	48 h	LC_50_(24h)/LC_50_(48h)	24h	48h	LC_50_(24h)/LC_50_(48h)
**3**	63.3 ± 3.2	33.0 ± 1.4	1.92	20.5 ± 3.0	17.2 ± 3.2	1.19
**4**	51.8 ± 2.5	36.2 ± 1.7	1.43	28.5 ± 3.7	27.3 ± 1.8	1.04
**5**	57.4 ± 7.9	43.9 ± 5.1	1.31	28.7 ± 2.1	29.2 ± 2.0	0.98

Results are expressed as mean ± SD. LC_50_; concentration that causes 50% lethality

At first, we investigated the effect of 10 kinds of polyphenols on cultured K562 and HEK293T cells. The cells were incubated with 100 μM polyphenol or 0.5 % DMSO (control) for 48 h (Figure 2). Cell viability was analyzed by quantitation of the adenosine triphosphate (ATP) present, that allowed direct comparison of living cell numbers between different polyphenols. Polyphenols **3**-**5** exhibited strong cytotoxicity, and while compounds **1**, **2**, **6**-**8** were not active (Figure 2). On the other hand, trimers **9** and **10** exhibited a weak but significant decrease in viable cells. We then analyzed effect of polyphenols **3**-**5** on K562 and HEK293T cell viability over a wide range of concentrations. The results are expressed as concentration that causes 50% lethality (LC_50_) (Table 1). Polyphenol **3** was the most toxic substance against K562 and HEK593T cells at 48 h with LC_50_ values of 33.0 and 17.2 μM, respectively. Each polyphenol **3**-**5** has one or more pyrogallol moiety, which were suggested to play an important role for cytotoxic activity. 

**Figure 3:**
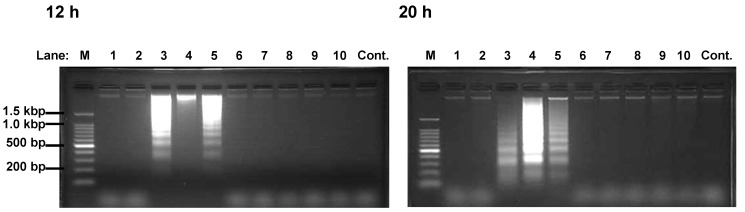
DNA fragmentation of HEK293T cells induced by Compound 3-5.

Cells were incubated with polyphenol **1**-**10** (Lane 1-10) or vehicle (Lane Cont.) for 12 h (left picture) or 20 h (right picture). DNA fragment extracts (8 x 10^5^ cells) were applied to 2.0% agarose gel containing 500 ng/mL ethidium bromide. Lane M is the DNA size marker. Similar results were obtained in four separate experiments.

Polyphenols **3** and **4** were reported as apoptosis-inducer against K562 [9,10]. Here, we investigated whether polyphenols containing pyrogallol moiety induced apoptosis against HEK293T. DNA ladders appeared in 12 and 20 h after incubation of the cells with polyphenols **3**-**5** (Figure 3). Since the appearance of the DNA fragmentation, which precedes the morphological change, is characteristic to apoptosis [11], the polyphenols **3**-**5** were considered to be apoptosis-inducers. These results indicated that the induction of apoptosis at least partly mediates their cytotoxic activity. In contrast, the rest of polyphenols did not induce DNA ladder formation. Thus, we concluded that compounds **9** and **10** cause decrease in viable cell through suppression of proliferation but induction of apoptosis (Table 1 and Figure 3). 

Polyphenols show strong anti-oxidative activity. To study the correlation between cytotoxicity and radical-scavenging activity, we analyzed radical-scavenging activity of polyphenols (**1**-**10**). 1‑Diphenyl-2-picryl-hydrazyl (DPPH) is a stable organic free radical with an absorption band in the range of 510-530 nm. The radical loses this absorption feature when accepting an electron or a free radical species, resulting in a visually noticeable discoloration from dark purple to pale yellow. Because the DPPH radical can accommodate many samples in a short time period and is sensitive enough to detect active ingredients at low concentrations, it has been extensively used to screen the anti-radical activities of compounds or plant extracts [12]. We examined the scavenging activity of the polyphenols on DPPH, and Trolox was used for a positive control. The concentrations that causes 50% effect (EC_50_) scavenging activity were calculated (Table 2). All polyphenols showed better DPPH scavenging activity than the positive control Trolox. Their scavenging abilities were classified in three groups and decreased in the following order: **4**, **9**, **10** > **3**, **5**-**8** > **1**, **2**. The superoxide anion (**^.^**O_2_**^–^**) is a major source of many free radicals. Hence, compounds that can scavenge the superoxide anion can reduce the production of many other radical species, such as peroxyl, alkoxyl, hydroxyl, and nitric oxide radical [13]. We evaluated the **^.^**O_2_**^–^** scavenging activity of the polyphenols using 2-methyl-6-*p*-methoxyphenylethynylimidazopyazinone (MPEC). The EC_50_ values of the **^.^**O_2_**^–^** scavenging activity were calculated (Table 2). The results indicated that all tested polyphenols showed an **^.^**O_2_**^–^** scavenging activity. According to EC_50_, their scavenging abilities were put in the following order: **4** > **3** > **5**, **9**, **10** > **2**, **6**, **7**, **8** > **1**. 

Although polyphenol **3** was the most potent cytotoxic compound, its radical scavenging activities against DPPH and **^.^**O_2_**^–^** were 0.67- and 0.57-fold weaker than those of polyphenol **4**. Importantly, anti-oxidative activities of the trimmers **9** and **10** were comparable to those of the pyrogallol-containing polyphenols (**3**-**5**), however, activities of cell death induction of **9** and **10** were obviously weaker than those of compounds **3**-**5** (Figure 2). Therefore, cytotoxicity of polyphenols cannot be fully explained by their antioxidative activities, and we suggest that pyrogallol moiety is important for fitting appropriately to their putative target molecule(s) in non-oxidative mechanism. 

**Table 2:** Radical scavenging activities of compounds.

Compound	EC_50_ (μM)	
	DPPH	^.^O_2_^–^
**1**	20.3 ± 0.2	123.4 ± 0.4
**2**	20.2 ± 0.3	83.3 ± 1.2
**3**	14.1 ± 0.2	12.3 ± 0.1
**4**	9.5 ± 0.2	7.0 ± 0.0
**5**	17.5 ± 0.2	23.9 ± 0.3
**6**	13.6 ± 0.1	84.7 ± 0.3
7	15.8 ± 0.2	91.1 ± 0.3
8	15.4 ± 0.1	74.2 ± 0.4
**9**	9.0 ± 0.0	26.8 ± 0.1
**10**	7.6 ± 0.0	24.0 ± 0.1
**Trolox**	43.6 ± 0.9	64.1 ± 0.2

Results are expressed as mean ± SD. EC_50_: concentration that causes 50% effect

## Experimental

### Reagents

Polyphenols **1**-**5** were purchased from Sigma (St Louis, MO, USA). Polyphenols **6**-**10** were synthesized as previously reported [14,15,16,17,18,19,20]. Polyphenols were dissolved in dimethyl sulfoxide (DMSO) and stored at –25 ^o^C. Trolox^®^, a water-soluble vitamin E analog, was purchased from Calbiochem (Darmstadt, Germany). MPEC was purchased from Atto (Tokyo, Japan). Other reagents were obtained from Wako Pure Chemical Industries, Ltd. (Osaka, Japan).

### Cell culture

The human chronic myelogenous leukemia cell line, K562 and human embryonic kidney (HEK) 293T cells were maintained in RPMI-1640 medium containing 10% fetal bovine serum, 1.9 g/L sodium bicarbonate, 100 μg/mL streptomycin, and 20 U/mL penicillin G at 37 ˚C under 5% CO_2_. 

### Determination of cytotoxicity

The cells were suspended in 100 μL of medium with various concentrations of polyphenol or 0.5% DMSO (vehicle) and plated in flat-bottom plates. K562 (1 x 10^5^ cells/mL) and HEK293T cells (2 x 10^5^ cells/mL) were cultured for 24 or 48 h, and then equal volume of CellTiter-Glo Reagent (Promega Corporation, Madison, WI, USA) was added. The CellTiter-Glo luminescent cell viability assay is a homogeneous method of determining the number of viable cells in culture based on quantitation of the ATP present. Chemiluminescence was determined by a microplate luminometer, Veritas^TM^ (Promega, Corporation).

### Assay for DNA fragmentation

HEK293T cells (8 x 10^5^ cells) were incubated with 100 μM polyphenol or 0.5% DMSO (control) in 2 ml of medium for indicated times. Treated-cells were washed with 1 mL phosphate buffer saline (‑)[PBS(-)] by centrifugation at 3,600 rpm, and then suspended in 40 μL of lysis buffer containing 10 μM Tris-HCl, pH7.5, 10 μM EDTA and 0.5% Triton X-100. The lysate was centrifuged at 13,000 rpm for 20 min. The supernatant was incubated with 20 μg RNase A at 37 ^o^C for 2 h. Then 2 μL of 20 mg/mL proteinase K was added and the mixture was incubated for 2 h. After the addition of 3 M NaCl (22 μL) and 2-propanol (66 μL), the mixture was allowed to stand overnight at –25 ^o^C. After centrifugation at 13,000 rpm for 20 min, the pellet was suspended with TE buffer (10 μM Tris-HCl, pH 8.0 and 1 μM EDTA, 16 μL), mixed with gel-loading buffer (40% sucrose, 1 μM EDTA and 0.25% Bromophenol Blue, 4 μL), and loaded on a 2% agarose gel. BEX 100 bp DNA Ladder MK (BEX CO., LTD., Tokyo, Japan) was used as DNA size marker. 

### Determination of DPPH radical scavenging activity

Various concentrations of polyphenols and Trolox^®^ as a positive control were prepared in 50% ethanol (HPLC grade). DPPH was dissolved in DMSO to 100 μM and stored at **-**25 ^o^C. Before use, 100 μM DPPH was diluted with ethanol to 444.4 μM, and further diluted with equal volume of water to give 222.2 μM DPPH solution. One-hundred-and-eighty μL of this solution was added to test samples (20 μL) in flat-bottom plates, and sealed with microplate sealing polypropylene tape (AS ONE, Osaka city, Osaka, Japan), and then incubated at room temperature for 35 min. The scavenging activity was estimated by measuring the absorption of the reaction mixture at 530 nm with the microplate reader (Tecan Trading AG, Männedorf, Switzerland). 

### Determination of superoxide anion scavenging activity

The superoxide anion (**^.^**O_2_**^–^**) scavenging ability of test samples was determined by MPEC system [13]. MPEC was dissolved in DMSO to 18 μM and stored at -80 ^o^C. Before use, 18 μM MPEC was diluted with ethanol to 4.5 μM, and further diluted with equal volume of water to 300 μM MPEC solution. Xanthine oxydase (Sigma) was diluted with buffer A (100 μM KH_2_PO_4_ [pH 7.5], 50 μM EDTA) containing 1 mg/mL BSA. Xanthine oxydase (0.2 U/mL, 30 μL) was mixed with MPEC (300 μM, 10 μL) and test samples (10 μL of various concentration) dissolved in water. The reaction was initiated with buffer A (250 μL) containing 144 μM hypoxanthine. Chemiluminescence was determined by a microplate luminometer, Veritas^TM^ (Promega, Corporation).

### Statistical analysis

Results are expressed as mean ± SD (n = 3) in Figure 2 and Table 1 and Table 2. Statistical differences were determined by one way-analysis of variance (ANOVA) followed by the Scheffe’s multiple comparison test. By the one-way ANOVA, a value of *p* < 0.05 was considered statistically significant. 
